# Changing the narratives for patient safety

**DOI:** 10.2471/BLT.16.178392

**Published:** 2017-04-25

**Authors:** Peter J Pronovost, Kathleen M Sutcliffe, Lopa Basu, Mary Dixon-Woods

**Affiliations:** a Johns Hopkins Medicine, Armstrong Institute for Patient Safety and Quality, Johns Hopkins University, 750 E Pratt Street, 15th floor, Baltimore, Maryland 21202, United States of America (USA).; b Johns Hopkins University, Carey Business School, Baltimore, Maryland, USA.; c Cambridge Centre for Health Services Research, University of Cambridge, Cambridge, England.

Patient safety is recognized as a global public health issue,^1^ causing death and suffering in all types of patients and incurring costs in all countries. The global health community has made significant and sustained efforts to improve safety and quality of health services. However, progress in reducing preventable harm has been too limited, little and local.^2^ Here, we propose that narratives or mental models are reasons for the limited progress. Narratives inform how we interpret reality and how to act in the world and those told about patient safety and poor quality care often inhibit rather than facilitate momentum to make changes.^3^ In this paper, we discuss how changing these narratives may accelerate the efforts to improve safety and quality of care.

One narrative is that patient harm is inevitable. There is limited systematic evidence, from a large number of countries, describing which harms are measured in health-care studies and which harms are the focuses of national policies to prevent them. Nevertheless, preventable harm has been identified as a significant problem in all care settings.^4^^,^^5^ While harm is usually focused on disabling injury or death from medical care, we also include here the less tangible harms, in which patients feel disrespected.

Nonetheless, a fatalistic story reinforces the status quo and frustrates efforts to better understand complex health-care systems and how to make them safer. In addition, assuming that harm is inevitable may partially explain the lack of national and global measures of patient harm, the widely varying estimates of the scope of the problem, and the gap between the scope of the problem and investments in resolving the problems. In particular, the belief that harm is inevitable hinders needed investments in transdisciplinary research to better understand what it means for a harm to be preventable in a complex context. The researchers also need to know the scale of the problem and what fundamental mechanisms are needed to improve safety and quality of care.

A second narrative is that clinicians are responsible for safety and their behaviours are the main targets for change. Yet evidence shows that no matter how hard an individual works to keep patients safe, poor systems may defeat them. Health-care organizations might have technologies with low usability and absent interoperability, underspecified work processes, immature and variable safety training, poorly developed management systems and opaque and ambiguous accountability mechanisms. Too often, clinicians work in systems that are not well designed and operationally are managed poorly. Too often, patient priorities and their experiences across the continuum-of-care are rarely considered when designing systems. Too often, policy-makers and managers execute extrinsic incentives instead of capitalizing on the intrinsic motivation of professionals. The policy-makers remain rooted in a hierarchical system rather than forming a system that balances independence and interdependence, enabling a foundation for improvement and valuing professional instincts.^6^

Health care is starting to change this narrative. Some notable improvements have occurred for hand hygiene adherence and bloodstream infections.^7^^,^^8^

A third narrative is that each organization should solve their patient safety problems alone. Individual organizations can achieve much – especially when they are highly intentional, committed and energetic – but single-organizational efforts can paradoxically introduce new risks by undermining standardization and coordination between hospitals and/or countries. However, when many people and institutional actors are involved in a collective activity, responsibility can be scattered and obscured. This might hinder large-scale coordination that is needed to solve many sorts of safety problems.^9^ Yet this challenge can be overcome by adapting strategies from other high-risk industries. Health-care researchers have studied the sector-wide, collective improvement efforts achieved in other high-risk industries, recognizing that systems engineering is needed to create integrated and holistic approaches to ensuring safety and reliability.^10^

A fourth narrative is that health care improvement will come by improving one process at a time (e.g. infections or blood clots) through bounded projects rather than designing an integrated system of operations to eliminate or reduce all harms. This narrative links to the first three, requiring a new narrative for integrated system improvement. Patients are all at risk for dozens of harms, many of them not clearly confined or easily targeted by highly specific efforts. What will likely be more effective is adaption of the safety management systems or operating management systems that high-risk industries use to integrate their approaches to safety and quality.^11^ The health-care sector is beginning to adopt high-reliability organizing principles from other industries.^12^

A fifth narrative is the role of patients in their safety. When they are included, it is often as victims (after the harm has occurred) or surveillance agents, supervising the behaviours of clinicians (e.g. asking the clinicians to wash their hands). Neither scenario is fair to patients, nor do these scenarios recognize the many settings in which patients are exposed to harm, including their own homes. Patients only spend a fraction of their time in health-care settings, but clinicians can facilitate patient safety outside these settings, for example, by helping to prevent falls and medication errors at home. Any such change should avoid transferring the responsibility to patients to keep themselves safe, whether they are in the hospital and particularly vulnerable, or at home.

Although these narratives are slowly changing, patient safety and quality of care need new narratives that liberate the constraints of current narratives and theories and emphasize the collective nature of the efforts required to learn and improve. These new narratives should also reveal the gap between the problems and current investments for solving them.

## Reframing narratives

To reshape these narratives, the global health community can increase research activities to better understand the extent to which a harm is preventable and which local- and sector-wide actions will reduce the harm. Research on which methods are most suitable for measuring harm and which mechanisms enable such measurements should also be increased. The World Health Organization (WHO) has adopted global resolutions, including World Health Assembly Resolution WHA55.18,^13^ to guide efforts to improve safety and quality of care. These resolutions alone will not reframe the old narratives into the new ones. Practical solutions to guide countries on how to achieve the ideal standards for safety and quality^13^ are essential to shift the global dialogue on preventing avoidable causes of human suffering.

One measurable step towards new narratives would involve developing standard measures for the major causes of patient harm, such as pressure ulcers, medication errors and diagnostic errors. These standards could be similar to the health-care associated-infections action plan made by the United States Centers for Disease Control and Prevention.^14^ Better measurement would enable estimates closer to the true size and scope of the problem and help resolve controversies about current claims about patient safety.

Second, the global health community could encourage coordination of sector-wide efforts to design safe work systems. Areas of focus could be better designed medical technologies and tools to reduce the cognitive load that can distract clinicians and lead to error, use of human factors-led interventions, and integration and interoperability of technological systems. Such efforts will require convening different disciplines to better understand how safer systems can be designed. A systems engineering approach, including transdisciplinary experts, starts with a goal, examines the purpose of the health-care system relative to the goal and then works backward to design a system to achieve that purpose. The approach aligns people, processes, technology and organizational climate to achieve the goal and needs leadership to align stakeholders around a common vision, coordinate efforts, provide resources and incentives and monitor progress. This is the approach other high reliability organizations, such as naval aircraft carriers, oil and gas companies, and nuclear power plants, improved. It is therefore encouraging that the WHO Framework on integrated people-centred health services^15^ is aligned with this systems approach, specifically by creating an enabling environment to strive for safety and quality improvement.

A third area of focus would encourage deeper, more holistic and theory-based learning from high-risk industries, rather than relying on single, superficial and siloed interventions. Such single interventions are usually implemented without fully understanding the supporting infrastructure required for them to work: an infrastructure that uses an integrated approach to investigate and then manage the multiple risks found in complex health systems. For example, some health-care safety researchers have borrowed ideas from the World Association of Nuclear Operators^16^ and started conducting peer-to-peer reviews to evaluate specific harms (e.g. infections), specific areas (e.g. operating rooms) or entire quality and safety programmes.^17^ These reviews are confidential, disciplined, deliberate and not part of a regulatory process that applies sanctions. Their focus is therefore on learning and sharing, not judging. Highly qualified and experienced technical experts perform the reviews, making them more likely to identify best practices and to be meaningful.^16^

A fourth step would be to engage academic institutions in low- and middle-income countries to build capacity for improving safety and quality in these countries. The model of global partnerships for global solidarity on quality and safety has already begun a new narrative, whereby human interaction across continents can drive change towards safety and quality of universal health coverage.^18^

Health care has made some progress in improving safety but work remains. However, no simple solutions exist. Much of the work involves enabling infrastructures that allow solutions to emerge. By reframing the narratives that guide our current approaches to patient safety, the global health community may be better at protecting patients against harm.
